# EqualTDRL: illustrating equivalent tandem duplication random loss rearrangements

**DOI:** 10.1186/s12859-018-2170-x

**Published:** 2018-05-30

**Authors:** Tom Hartmann, Matthias Bernt, Martin Middendorf

**Affiliations:** 1 Swarm Intelligence and Complex Systems Group, Faculty of Mathematics and Computer Science, Leipzig University, Augustusplatz 10, Leipzig, D-04109 Germany; 2 Helmholtz Centre for Environmental Research - UFZ, Permoserstraße 15, Leipzig, D-04318 Germany

**Keywords:** Circular permutation, Gene order, Genome rearrangement, Mitochondria, Tandem duplication random loss

## Abstract

**Background:**

To study the differences between two unichromosomal circular genomes, e.g., mitochondrial genomes, under the tandem duplication random loss (TDRL) rearrangement it is important to consider the whole set of potential TDRL rearrangement events that could have taken place. The reason is that for two given circular gene orders there can exist different TDRL rearrangements that transform one of the gene orders into the other. Hence, a TDRL event cannot always be reconstructed only from the knowledge of the circular gene order before a TDRL event and the circular gene order after it.

**Results:**

We present the program EqualTDRL that computes and illustrates the complete set of TDRLs for pairs of circular gene orders that differ by only one TDRL. EqualTDRL considers the circularity of the given genomes and certain restrictions on the TDRL rearrangements. Examples for the latter are sequences of genes that have to be conserved during a TDRL or pairs of genes that frame intergenic regions which might represent remnants of duplicated genes. Additionally, EqualTDRL allows to determine the set of TDRLs that are minimum with respect to the number of duplicated genes.

**Conclusion:**

EqualTDRL supports scientists to study the complete set of TDRLs that possibly could have taken place in the evolution of mitochondrial genomes. EqualTDRL is implemented in C++ using the ggplot2 package of the open source programming language R and is freely available from http://pacosy.informatik.uni-leipzig.de/equaltdrl.

**Electronic supplementary material:**

The online version of this article (10.1186/s12859-018-2170-x) contains supplementary material, which is available to authorized users.

## Background

The genetic information of species is stored in DNA (or RNA) molecules. These molecules are called chromosomes and can either be *linear* or *circular*. The set of all these molecules of a species forms its *genome*. The genome consists of *genes* which are DNA segments with certain functions. Mutations can modify the arrangement or the multiplicity of the genes within the genome. Such mutations are called *rearrangements*. The research field of genome rearrangement analysis tries to explain the differences between two genomes that are represented by the arrangement of their genes, in order to infer phylogenetic information. The following two central problems exist in this research field [1]. The *distance problem* aims to determine the *distance* between two genomes, i.e., the smallest number of rearrangements (of certain types) that are needed to transform one gene order into the other. The *sorting problem* asks for a shortest sequence of rearrangements for such a transformation. Such a sequence is called a *shortest scenario*. In case that costs can be assigned to different types of rearrangements the distance problem (the sorting problem) asks for the minimum total cost for the transformation from one gene order to the other (respectively, for a corresponding minimum cost sequence of rearrangements).

One important type of rearrangements is the *tandem duplication random loss* (TDRL) rearrangement. A TDRL consists of a tandem duplication of a contiguous set of genes followed by a random loss of one copy of each duplicated gene. TDRLs have occurred several times in the evolution of mitochondrial genomes, e.g., in gulper eels [2] and in millipedes [3]. Several mechanisms that explain a TDRL have been discussed in the biological literature, e.g., slipped strand mispairing during replication or imprecise termination [4]. It has been shown that TDRLs are a major factor of gene order evolution for mitochondrial genomes [4–6].

The TDRL rearrangement was initially studied formally for linear genomes in [7]. The cost of a TDRL rearrangement is defined as *α* ^*k*^, where *α*≥1 is a parameter and $k\in \mathbb {N}$ is the number of genes that are influenced by the TDRL. For the cases *α*=1 and *α*≥2 polynomial time algorithms that solve the sorting problem (and therefore the distance problem) have been presented in [7]. In addition, it has been shown for *α*=1, that it is sufficient to consider TDRLs that duplicate the whole genome, because the cost of every TDRL is the same in this case. Here we consider the case *α*=1 as well. Note that this definition of a TDRL considers explicitly only whole genome duplications but implicitly also partial genome duplications, as explained in detail in “Implementation” section.

The authors of [3] studied an alternative version of TDRL rearrangement where genes with the same orientation or genes that belong to the same transcript are lost jointly, i.e., the loss is not completely random but depends on gene orientation or transcript structure.

In order to reconstruct genome rearrangements reliably it is important to consider the possibility of alternative shortest rearrangement scenarios and equivalent rearrangements, i.e., rearrangements that when applied to the same gene order lead also to the same resulting gene order. This has been studied for the inversion, the transposition and the double cut and join (DCJ) rearrangement [8, 9]. Equivalent inversions for signed circular permutations, which represent circular genomes, have been discussed in [10] and all shortest scenarios for the sorting of signed permutations have been studied in depth in [11–14]. Since every transposition can be represented by a TDRL, the set of equivalent transpositions has been studied in [15]. The set of all shortest scenarios of the problem to sort a multichromosomal genome by DCJ rearrangements has been investigated in [16]. In this work the authors also determined the exact number of optimal shortest scenarios for a particular set of problem instances.

An analysis of TDRL rearrangements on circular genomes has been presented in [15]. It has been shown that the circularity of the genomes should be considered, since the TDRL distance for an unfavorable choice of linear representatives (of the circular genomes) may lead to an overestimation of the distance. In addition, it has been shown that it is not always possible to uniquely reconstruct a TDRL only from the knowledge of the two circular gene orders before and after the application of the TDRL because there exist several TDRLs that can explain the change from one circular genome to the other.

Software tools that regard TDRL rearrangements for the construction of evolutionary scenarios are CREx [17], CREx2 [18], and TreeREx [19]. However, these tools present only a single shortest scenario in the case that a TDRL is present in a shortest scenario. Hence, when a scenario contains a TDRL, the tools do not show all possible alternative TDRLs. Therefore, it is important to further analyze the solutions that are generated and the software EqualTDRL that we present in this paper is designed to support scientists to do such an analysis.

EqualTDRL uses results from [15] to consider the circularity of the given gene orders. It provides figures that illustrate all equivalent TDRL operations for two given gene orders that differ by only a single TDRL. Therefore, EqualTDRL supports scientists to study the whole set of TDRLs that possibly could have taken place. This is beneficial in several aspects of inferring a most reliable TDRL. For example: i) for identifying whether the gene loss of a TDRL is random or is dependent on gene orientation or transcript structure, ii) for finding a (partial duplication) TDRL that duplicates only a minimum number of genes, iii) to determine a subset of TDRLs that satisfy certain conditions, e.g., to preserve certain sequences of genes that cannot be broken during a TDRL, and iv) for identifying the positions of potential duplication remnants for a further analyses of the nucleotide sequence.

This article is organized as follows. In the next “Implementation” section a formal background is given on circular gene orders and TDRL rearrangements. Further, an overview on EqualTDRL is presented. In “Results and discussion” section the benefits of EqualTDRL are shown for a biological example of mitochondrial gene orders. The article ends with a conclusion in “Conclusion” section.

## Implementation

### Methods

**Fig. 1:**
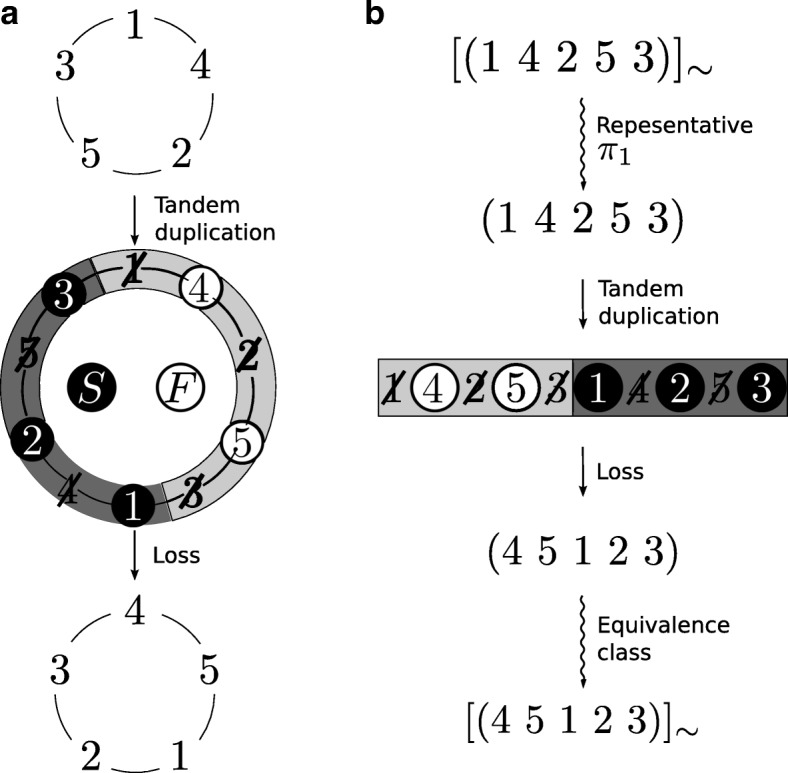
Application of TDRL ({4,5},{1,2,3},1) to the circular permutation [(1 4 2 5 3)]_∼_. The resulting circular permutation is [(4 5 1 2 3)]_∼_. The circular permutation [(1 4 2 5 3)]_∼_ (respectively [(4 5 1 2 3)]_∼_) is represented in (**a**) by a circular illustrations on the top (respectively bottom) which gives the corresponding representatives when read in clockwise direction. Whereas (**a**) shows the application of the TDRL by using circular illustrations, (**b**) illustrates the same application by applying a tandem duplication to the representative *π*_1_∈[(1 4 2 5 3)]_∼_ followed by the subsequent loss of one copy of every duplicated gene. In particular, the elements of *F*={4,5} are kept in the first copy (illustrated by a bright gray) and the elements of *S*={1,2,3} are kept in the second copy (illustrated by a dark gray) of the duplicated intermediate. Elements that are lost during this process are crossed out. Permutation *τ*∘*π*_1_=(4 5 1 2 3) is a representative of the resulting circular permutation, namely [*τ*∘*π*_1_]_∼_=[(4 5 1 2 3)]_∼_

In this article it is assumed that the genes in a genome (before and after a TDRL) are not duplicated and that genes do not overlap. Therefore, a gene order of a (circular) genome can be represented by a (circular) permutation. A *permutation* *π* of length *n*, denoted by *π*=(*π*(1) … *π*(*n*)), is a bijection *π*:[1:*n*]→[1:*n*]. The set of all permutations of length *n* is denoted by $\mathcal {S}_{n}$. The *shift operation* $\phi \colon \mathcal {S}_{n} \rightarrow \mathcal {S}_{n}$ is defined by *π*=(*π*(1) … *π*(*n*))↦(*π*(2) … *π*(*n*) *π*(1)) and for $k \in \mathbb {N}_{>0}$ the *k-shift* is *ϕ* ^*k*^∘*π*, where *ϕ* ^*k*^:=*ϕ*∘*ϕ* ^*k*−1^ and *ϕ* ^1^:=*ϕ*. Note that with *f*∘*g* the composition of two functions *f* and *g* is denoted, i.e., (*f*∘*g*)(*x*):=*f*(*g*(*x*)). With ∼ we denote the equivalence relation on $\mathcal {S}_{n}$, where $\pi,\pi '\in \mathcal {S}_{n}$ are equivalent, denoted by *π*∼*π* ^′^, if and only if there exists an *m*∈[1:*n*] with *ϕ* ^*m*^(*π*)=*π* ^′^. The equivalence class *π*°:=[*π*]_∼_ of ∼ on $\mathcal {S}_{n}$ is called a *circular permutation* of length *n* and the set of all circular permutations of length *n* is denoted by $\mathcal {S}^{\circ}_{n}$. In other words, a circular permutation *π*°∈*S*°_*n*_ is the set of all permutations that are equivalent with respect to ∼, i.e., *π*°={*π*,*ρ*(*π*),…,*ρ* ^*n*−1^(*π*)} or, less formally, a circular permutation is the set of all permutations that become equivalent when the first and the last element of a permutation are considered to be adjacent. Figure 1a shows an illustration of a circular permutation. Each *π*∈*π*° is called a *representative* of *π*° and the representative which starts with element *p*∈[1:*n*] is denoted by *π*_*p*_. Circular permutations are used as a formal model for unichromosomal circular genomes, e.g., mitochondrial genomes, in which each element represents a gene and each representative stands for a possible linearization of the considered genome.

A TDRL *τ*°:*S*°_*n*_→*S*_*n*_° is a bijection that is denoted by a triple (*F*,*S*,*p*), where (*F*,*S*) is a bipartition of [1:*n*], i.e., *F*,*S*⊂[1:*n*], *F*∩*S*=*∅*, and *F*∪*S*=[1:*n*], and *p*∈[1:*n*]. Element *p* is called the *origin* of (*F*,*S*,*p*) and it denotes the position where the whole genome duplication of *π*° starts resulting in a duplicated intermediate. The sets *F* and *S* are a bipartition of [1:*n*] and denote the sets of genes that are kept (i.e., they are not deleted) in the first and second copy of *π*_*p*_ in the duplicated intermediate, respectively. Here “first” and “second” are defined with respect to *π*_*p*_. The elements that are deleted in the duplicated intermediate are called *lost elements*. The effect of a TDRL *τ*°=(*F*,*S*,*p*) on *π*° is defined as *τ*°∘*π*°:=[*τ*∘*π*_*p*_]_∼_, where *τ*∘*π*_*p*_ is a rearranged permutation of *π*_*p*_ such that the elements of *F* are moved in front of the elements of *S* and the relative order of all elements of *F* (respectively *S*) of *π*_*p*_ is unchanged. More precisely, *τ*∘*π*_*p*_ is the (linear) permutation for which it holds that 1) if *i*∈*F*, *j*∈*S*, then *i* is to the left of *j* in *τ*∘*π*_*p*_ and 2) if *i*,*j*∈*F* or *i*,*j*∈*S*, then *i* is to the left of *j* in *τ*∘*π*_*p*_ if and only if *i* is to the left of *j* in *π*_*p*_. Note that by this definition, a TDRL (*F*,*S*,*p*), which maps a set *π*° of permutations to another set of permutations, can be visualized by reordering the elements of the representative *π*_*p*_ according to the sets *F* and *S*, see Fig. 1b for an example. For the combinatorics of TDRLs it is irrelevant whether *F* or *S* is the set of genes that are preserved in the original part of the duplicated intermediate, since both applications result in the same circular permutation [15], i.e., (*F*,*S*,*p*)∘*π*°=(*S*,*F*,*p*)∘*π*°. Note that the strandedness of a gene (also called its orientation) is not relevant for this paper since a TDRL does not change the strandedness of genes. This is also the reason why we represent a gene order as an (unsigned) permutation.

Two TDRLs are called *equivalent* if their application to the same (circular) permutation results in the same (circular) permutation. It was shown that the number of equivalent TDRLs is 2*n* ^2^ if the TDRLs are identity maps and 2*n* otherwise, where *n* is the length of permutation *π*° [15]. The *TDRL distance* of *π*° and *σ*° is $d(\pi^{\circ},\sigma^{\circ})=\min (\{d\in \mathbb {N}_{0}\vert \exists \text { TDRLs }\tau^{\circ}_{1},\ldots,\tau^{\circ}_{d}: \tau^{\circ}_{d}\circ \ldots \circ \tau^{\circ}_{1}\circ \pi^{\circ}=\sigma^{\circ}\})$.

The definitions related to TDRL rearrangements and circular permutations are exemplified for the circular permutation *π*°=[(1 4 2 5 3)]_∼_. See Fig. 1 for an illustration. The representatives of *π*° are *π*_1_=(1 4 2 5 3), *π*_2_=(2 5 3 1 4), *π*_3_=(3 1 4 2 5), *π*_4_=(4 2 5 3 1), and *π*_5_=(5 3 1 4 2), i.e., *π*°={*π*_1_,…,*π*_5_}. It holds that *π*_2_=*ϕ* ^2^(*π*_1_), *π*_3_=*ϕ* ^2^(*π*_2_), *π*_4_=*ϕ* ^2^(*π*_3_), *π*_5_=*ϕ* ^2^(*π*_4_), and *π*_1_=*ϕ* ^2^(*π*_5_). The application of TDRL *τ*°=({4,5},{1,2,3},1) to *π*° gives [(4 5 1 2 3)]_∼_. Since genes 1 and 2 are in *S* and 1 is to the left of 2 in *π*_1_, it holds that 1 is to the left of 2 in (4 5 1 2 3). Also, since 5∈*F* and 3∈*S*, it holds that 3 is to the right of 5 in (4 5 1 2 3). Figure 1 illustrates the application of *τ*° to [(1 4 2 5 3)]_∼_. Since ({3,4},{1,2,5},5)∘*π*°=({4,5},{1,2,3},1)∘*π*° it holds that TDRLs ({3,4},{1,2,5},5) and ({4,5},{1,2,3},1) are equivalent.

**Fig. 2:**
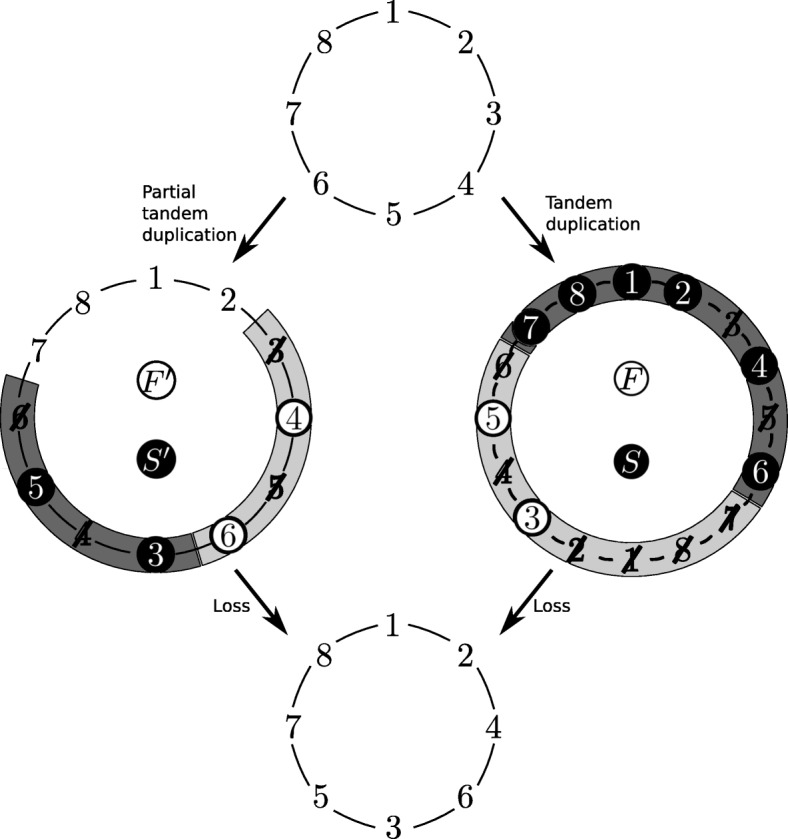
Partial duplication TDRL and a corresponding TDRL that achieves the same circular output permutation [(1 2 4 6 3 5 7 8)]_∼_. Notation is as in Fig. 1. The left-hand side shows a partial tandem duplication of the sequence 3 4 5 6 of [(1 2 3 4 5 6 7 8)]_∼_ followed by a subsequent loss of the elements 3 and 5 in the first copy, and 4 and 6 in the second copy, i.e., *F* ^′^={4,6}, *S* ^′^={3,5}, and the elements of *N*={1,2,7,8} are not duplicated. The same rearrangement can be achieved by the TDRL (*F*,*S*,*p*), where *F*=*S* ^′^, *S*=*N*∪*F* ^′^, and *p*=7 is the unique non-duplicated element adjacent to the second copy. The corresponding TDRL ({3,5},{1,2,4,6,7,8},7) is illustrated on the right-hand side

To see that the formal model also covers partial duplication TDRLs (i.e., TDRLs were not all genes are duplicated) consider a partial duplication TDRL that transforms *π*° into *σ*°. For every element *e*∈[1:*n*] it holds that either *e*∈*F* ^′^ (i.e., *e* is kept in the first copy), *e*∈*S* ^′^ (i.e., *e* is kept in the second copy), or *e*∈*N* (i.e., *e* is not duplicated). Then the TDRL (*F*,*S*,*p*), where *F*=*S* ^′^, *S*=*N*∪*F* ^′^, and *p* being the unique non-duplicated element adjacent to one element of the second copy in the partially duplicated intermediate, gives the same circular output permutation (see Fig. 2 for an example). Consequently, the same rearrangement can be achieved by a TDRL that duplicates all elements [15].

### EqualTDRL

**Fig. 3:**
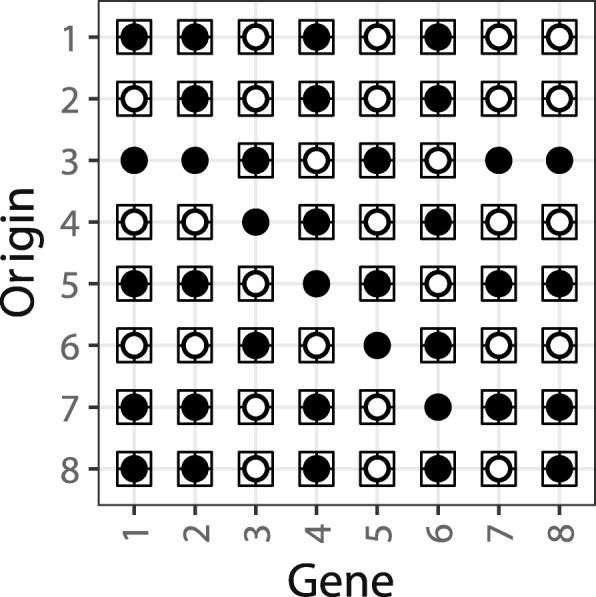
Output created by EqualTDRL: all TDRL rearrangements that transform [(1 2 3 4 5 6 7 8)]_∼_ into [(1 2 4 6 3 5 7 8)]_∼_. Each row illustrates the TDRL rearrangements (*F*,*S*,*p*), where *p* is the origin (y-axis) and a gene (x-axis) is in the set *F* (respectively *S*) if the corresponding circle is filled with white colour (respectively black colour). A square surrounding a circle shows that the corresponding gene is part of the duplicated sequence of the corresponding partial duplication TDRL

The software tool EqualTDRL calculates for two circular permutations *π*° and *σ*° that represent two circular unichromosomal genomes the distances *d*(*π*°,*σ*°) and *d*(*σ*°,*π*°). In the case that *d*(*π*°,*σ*°)=1 (respectively *d*(*σ*°,*π*°)=1) EqualTDRL produces an illustration which shows all equivalent TDRL rearrangements, i.e., all triples (*F*,*S*,*p*) that transform *π*° into *σ*° or vice versa. An example of such an illustration is given in Fig. 3 for the circular permutations *ι*°=[(1 2 3 4 5 6 7 8)]_∼_ and *π*°=[(1 2 4 6 3 5 7 8)]_∼_ with *d*(*ι*°,*π*°)=1. Figure 3 illustrates all equivalent TDRLs that transform *ι*° into *π*°:({3,5,7,8},{1,2,4,6},1), ({1,3,5,7,8},{2,4,6},2),({4,6},{1,2,3,5,7,8},3),({1,2,5,7,8},{3,4,6},4),({3,6},{1,2,4,5,7,8},5),({1,2,4,7,8},{3,5,6},6), ({3, 5},{1,2,4,6,7,8},7), ({3,5,7},{1,2,4,6,8},8), and all TDRLs that can be obtained from the listed TDRLs by interchanging the sets *F* and *S*. Recall that TDRL ({3,5},{1,2,4,6,7,8},7) is illustrated in Fig. 2.

Moreover, EqualTDRL can illustrate all partial TDRLs in addition to the whole genome TDRLs. This is shown in Fig. 3 where a black square surrounds the circle of a gene if and only if the gene is part of the duplicated sequence of a partial duplication TDRL. Note that for partial TDRLs it is not important whether genes that are excluded from the duplication are considered to be in *F* or in *S* since they are not duplicated and therefore no copy of these genes is lost. As an example, consider the origin 3 in Fig. 3. The figure shows that TDRL ({4,6},{1,2,3,5,7,8},3), which considers the whole permutation to be duplicated, can be replaced by a partial duplication TDRL that only duplicates the sequence 3 4 5 6 and contains the genes 4, 6 in the – with respect to the origin – first copy of the duplicated intermediate and genes 3, 5 in the second copy of the duplicated intermediate. See Fig. 2 for an illustration of the specified partial duplication TDRL and a corresponding TDRL that duplicates the whole permutation.

Finally, EqualTDRL is also able to illustrate only those TDRLs that satisfy the following types of conditions which can be given by the user: i) specific sets of sequences of genes that are conserved by a TDRL and ii) intergenic regions that are framed by specific pairs of genes. Both conditions and the type of input that they require are explained in the following.

For the first type of condition the user has to specify the corresponding sets of genes. Then, EqualTDRL proceeds as explained in the following. Consider a set of genes *G*⊂[1:*n*], two circular permutations *π*° and *σ*° such that *d*(*π*°,*σ*°)=1, and a TDRL *τ*°=(*F*,*S*,*p*) with *τ*°∘*π*°=*σ*°. When *G* is used as a condition EqualTDRL only considers equivalent TDRLs (*F* ^′^,*S* ^′^,*p* ^′^) of *τ*° such that either *G*⊆*F* ^′^ or *G*⊆*S* ^′^. Therefore, if there exist such an equivalent TDRL all genes of *G* are lost in the same copy and the loss of this TDRL depends on *G*. In the case that set *G* contains exactly all genes that belong to the same transcript the first type of condition is used to determine whether or not the loss of a TDRL depends on a given transcript structure. Note that EqualTDRL can also use multiple gene sets. For an example consider Fig. 3 that illustrates all equivalent TDRLs that transform *ι*°=[(1 2 3 4 5 6 7 8)]_∼_ into *π*°=[(1 2 4 6 3 5 7 8)]_∼_. Let 1 2 and 7 8 be a sequence of genes (of *π*° and *ι*°) that shall be conserved by a TDRL, hence *G*_1_={1,2} and *G*_2_={7,8}. If these conditions are specified, then EqualTDRL provides an illustration similar to Fig. 3 but with the difference that it does not show the TDRLs for origin 2 and 8, i.e., ({1,3,5,7,8},{2,4,6},2) and ({3,5,7},{1,2,4,6,8},8). These TDRLs are not illustrated since for *i*∈[1:2] neither *G*_*i*_⊆*F* nor *G*_*i*_⊆*S*. However, for all other origins *p*∈{1,3,4,5,6,7} it holds that *G*_1_ and *G*_2_ are subsets of either *F* or *S*.

The second type of condition requires to find all possible TDRLs that allow to deduce intergenic regions between pairs of genes that are given to EqualTDRL as an input from the user. To see this, consider two circular permutations *π*° and *σ*°, a TDRL *τ*°=(*F*,*S*,*p*) such that *τ*°∘*π*°=*σ*°, and a pair of two distinct genes *x*,*y*∈[1,*n*] that is given by the user. Such a pair of genes should frame an intergenic region in the genome that is represented by *σ*°. Then EqualTDRL considers only equivalent TDRLs (*F* ^′^,*S* ^′^,*p* ^′^) of *τ*° where at least one gene of the duplicated intermediate that is with respect to $\pi _{p'}\phantom {\dot {i}\!}$ between *x* and *y* is deleted, i.e., there exists at least one gene *z*∈[1:*n*]∖{*x*,*y*} such that *z* is between *x* and *y* in the duplicated intermediate, *z* is lost, and either *x*∈*F* and *y*∈*S* or if *x*,*y*∈*F* (respectively *x*,*y*∈*S*) then *z*∈*S* (respectively *z*∈*F*). For such a TDRL the intergenic region between *x* and *y* in the genome that is represented by *σ*° can be explained by an incomplete deletion of the gene (or the set of genes) that were lost between *x* and *y*. Therefore, if such a TDRL exists EqualTDRL provides evidence that an intergenic region might be a remnant of a gene (or the set of genes) that is formed by an incomplete deletion of the same. For an example consider Fig. 3 that illustrates all equivalent TDRLs that transform *ι*°=[(1 2 3 4 5 6 7 8)]_∼_ into *π*°=[(1 2 4 6 3 5 7 8)]_∼_. Assume that an intergenic region is between genes 3 and 5 in *π*° and that one is interested to know which TDRLs (and which corresponding gene losses) applied to *ι*° could possibly result into this arrangement. Hence *x*=3 and *y*=5 are chosen. If this condition is given, EqualTDRL would produce Fig. 3. Hence, all illustrated TDRLs allow to deduce the intergenic region between 3 and 5. This holds because in all TDRLs of Fig. 3 either 3∈*F* and 5∈*S* (e.g., origin 5), 5∈*F* and 3∈*S* (e.g., origin 4), or 3,5∈*F* (respectively 3,5∈*S*) and the gene 4, which is between 3 and 5 in the duplicated intermediate, is lost. If, for example, condition *x*=1 and *y*=2 is used, then EqualTDRL provides a figure that illustrates only TDRL ({1,3,5,7,8},{2,4,6},2) of Fig. 3 (i.e., the row of Fig. 3 with origin 2). This holds since 1∈*F*, 2∈*S*, and 3 is between 1 and 2 in *π*_2_. For all other origins *p*∈[1:8]∖{2} holds that 1,2∈*F* (respectively 1,2∈*S*) and there does not exist an element *z* between 1 and 2 in the duplicated intermediate that is lost.

## Results and discussion

### Experiment

**Fig. 4:**
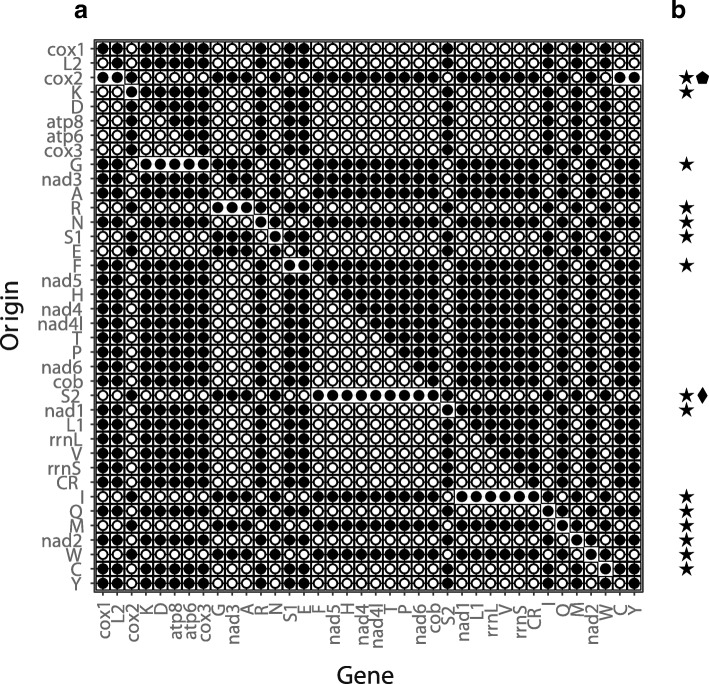
**a** Output created by EqualTDRL: complete set of TDRLs that rearrange the mitochondrial gene order of *Prionoglaris stygia* into the mitochondrial gene order of *Lepidopsocidae sp*. Protein-coding and ribosomal genes are denoted by their names and one capital letter indicates the amino acid for the tRNAs. **b** A star denotes a TDRL that does not break a conserved gene sequence. A pentagon highlights the TDRL that has been presented in [20]. A diamond highlights the TDRL which duplicates the minimum number of genes (the corresponding minimum TDRL is illustrated in Fig. 5)

In this section we show on the basis of mitochondrial gene orders how EqualTDRL can be used to find a plausible TDRL rearrangement. Specifically, the following two mitochondrial gene orders are considered: i) the gene order of *Prionoglaris stygia* (Genbank, accession: MG255141.1), which represents the ancestral mitochondrial gene order of the *Pancrustacea* [20], and ii) the mitochondrial gene order of the Trogiomorpha species *Lepidopsocidae sp.* (RefSeq, accession: NC _004816.1) and *Dorypteryx domestica* (Genbank, accession: MG255136.1) that has been published at the NCBI RefSeq release 84 [21] and the NCBI Genbank release 221 [22]. Note that the mitochondrial genomes of *Lepidopsocidae sp.* and *Dorypteryx domestica* comprise the same gene order. Both gene orders have recently been discussed in [20]. In this publication the authors studied the phylogenetic relationships between different barklice species of *Psocoptera* (a taxon which includes all considered species) and estimated the history of rearrangements using the condition-based coding algorithm MacClade [23], which does not support TDRL rearrangements, and the TreeREx software [19]. For both given mitochondrial gene orders the TreeREx analysis resulted in a large TDRL rearrangement which transforms the gene order of *Prionoglaris stygia* into the gene order of *Lepidopsocidae sp.* by duplicating 34 of 38 genetic markers (37 genes plus the control region). The corresponding TDRL is illustrated in Fig. 4 for the origin *cox2*.

Since for circular gene orders several equivalent TDRLs exist [15], we investigate the set of all possible TDRL rearrangements in the following. Since we also want to consider the intergenic sequences the mitochondrial genomes of the Trogiomorpha species *Lepidopsocidae sp.* and *Dorypteryx domestica* have been reannotated with an extended version of MITOS (unpublished http://mitos2.bioinf.uni-leipzig.de) [24]. In order to examine whether an intergenic region contains remnants of genes caused by an incomplete gene loss, the following data analysis was executed for *Lepidopsocidae sp.* and *Dorypteryx domestica*. For every tRNA and ribosomal gene (respectively protein-coding gene) the Covariance Models (respectively Hidden Markov Models) that are used in MITOS were applied i) to search in every intergenic region for every gene sequence and ii) if a gene (or a remnant of a gene) has been found to (locally) align the gene sequence to the corresponding intergenic region. The analysis was carried out for the tRNA and ribosomal genes with CMsearch and CMalign from the Infernal 1.1rc4 software package [25] and for protein-coding genes with HMMsearch and HMMalign from the HMMER 3.1b1 software package [26]. Moreover, for both mitochondrial gene orders the conserved sequences of genes (i.e., the maximal – with respect to inclusion – sequences of genes that occur in both gene orders) were calculated.

Then EqualTDRL was used to determine TDRLs that transform the mitochondrial gene order of *Prionoglaris stygia* into the mitochondrial gene order of *Lepidopsocidae sp.* under four different objectives: A) to show all possible TDRLs, B) to show all TDRLs that do not break any conserved sequence of genes, C) to highlight a subset of the TDRLs of (B) that provide additional evidence for the origin of intergenic regions outside of conserved sequences by an incomplete gene loss, and D) to find all TDRLs that minimize the number of duplicated genes.

### Results

The gene orders which result from the annotation that has been done with MITOS for the mitochondrial genomes of *Prionoglaris stygia*, *Lepidopsocidae sp.*, and *Dorypteryx domestica* are equal to the gene orders that have been discussed in [20].

**Fig. 5:**
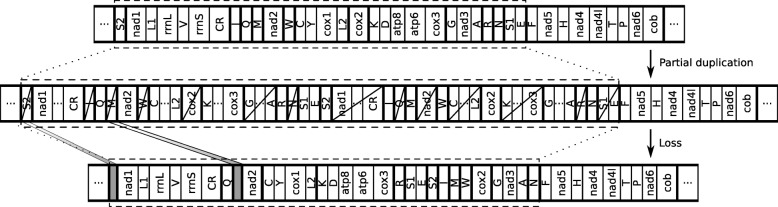
TDRL with a minimum number of duplicated genes that rearranges the mitochondrial gene order of *Prionoglaris stygia* (top) via the gene order after the duplication (middle) into the gene order of *Lepidopsocidae sp.* (bottom). Sequences that are bounded by a thick black line are conserved in both gene orders. Horizontal dots indicate the circularity of the gene orders. Intergenic regions of *Lepidopsocidae sp.* are indicated by gray squares. The sequence of genes that is rearranged is framed by a dashed square. Gene abbreviations and notation as in Fig. 4

A linear representation of the mitochondrial gene orders of *Prionoglaris stygia* and *Lepidopsocidae sp.* (respectively *Dorypteryx domestica*) that are used in this study is shown in Fig. 5. It can be seen that both gene orders contain the following conserved sequences: i) *trnC* *trnY* *cox1* *trnL2*, ii) *trnK* *trnD* *atp8* *atp6* *cox3*, iii) *trnG* *nad3* *trnA*, iv) *trnS1* *trnE*, v) *trnF* *nad5* *trnH* *nad4* *nad4l* *trnT* *trnP* *nad6* *cob*, and vi) *nad1* *trnL1* *rrnL* *trnV* *rrnS* *CR*, where *CR* denotes the control region.

The analysis with MITOS shows that the genome of *Lepidopsocidae sp.* contains 10 intergenic regions with a length of at least 10 base pairs, whereby only the three regions between *trnQ* and *nad2* (79 base pairs), *nad2* and *trnC* (105 base pairs), and *cob* and *nad1* (58 base pairs) are not contained in a conserved sequence. It is worth to mention that the intergenic region between *nad2* and *trnC* cannot be found in a less restrictive annotation with MITOS (when E-value exponent of 1 is used instead of the default value 2 for BLAST [27] and CMsearch). In this less restrictive annotation the putative intergenic region is completely assigned to *nad2*. The mitochondrial genome of *Dorypteryx domestica* contains 4 intergenic regions with a length of at least 10 base pairs: between *trnQ* and *nad2* (50 base pairs), *trnC* and *trnY* (31 base pairs), *trnN* and *trnF* (25 base pairs), and *cob* and *nad1* (21 base pairs). Note that the intergenic region between *trnC* and *trnY* is contained in a conserved interval. Since *Lepidopsocidae sp.* and *Dorypteryx domestica* have the same gene order, which is different to the ancestral *Pancrustacea* gene order, we assume that the TDRL studied in the following has happened before the speciation of the Trogiomorpha species. Therefore, we consider only intergenic regions that occur in both mitochondrial genomes of *Lepidopsocidae sp.* and *Dorypteryx domestica*. Thus in the analysis that follows we consider the intergenic regions between *trnQ* and *nad2*, and between *cob* and *nad1*. These intergenic regions are particularly interesting for further analysis since the presence of a gene remnant in those intergenic regions can be used to identify the subset of TDRLs that can explain the presence of the remnants as a result of an incomplete gene loss of a TDRL.

Figure 4a was generated with EqualTDRL and shows all equivalent TDRLs that transform the gene order of *Prionoglaris stygia* into the gene order of *Lepidopsocidae sp*. It also shows the set of duplicated genes for every TDRL. In Fig. 4b symbols were (manually) added to the right of a TDRL if and only if the TDRL exhibits specific characteristics: those that do not break any of the conserved sequences of genes and those that supports conjectures that can explain the presence of the intergenic regions as a result of an incomplete gene loss. These indications can be used to further search for gene remnants. Interestingly, it can be seen in Fig. 4 that the TDRL which has been presented in [20] preserves all conserved sequences of genes and it also explains the presence of both considered intergenic regions.

The HMMER and Infernal software package were used to identify potential remnants of genes. The experiments showed that *trnM* and *trnS1* can be found in the intergenic region of *Lepidopsocidae sp.* between *trnQ* and *nad2* with an E-value of 0.005 and 0.016, respectively. In addition several protein-coding genes hit the intergenic region between *trnQ* and *nad2*. However, the E-values of these hits are larger than 0.2 and therefore less reliable. Interestingly, no hit with an E-value smaller than 1 was found for both intergenic regions of *Dorypteryx domestica* and the intergenic region between *cob* and *nad1* of *Lepidopsocidae sp*. A table that summarizes all hits in the intergenic regions, its E-values, and the corresponding alignments can be found in the Additional file 1: Table S3 and Figures S1–S10.

Figure 4 shows that there exists a TDRL which duplicates only 29 of 38 genetic markers, which are 5 less than the TDRL that has been presented in [20]. Interestingly, this TDRL also preserves all conserved gene sequences and provides evidence for the presence of the intergenic regions regions between *trnQ* and *nad2*, and *cob* and *nad1* as a result of an incomplete gene loss of the genes *trnM* and *trnS2*, respectively. While the origin of the former intergenic region by the loss of *trnM* is supported weakly by our analysis, the origin of the latter intergenic region has no support from our sequence/structural similarity based analysis. The corresponding TDRL is illustrated in Fig. 5.

Altogether, the scenario presented in [20] partially agrees with the results that are presented in this article. However, due to the circularity of the mitochondrial gene orders it is important to consider the complete set of TDRL rearrangements that can explain the gene order of *Lepidopsocidae sp*. When it is considered to be relevant that a TDRL duplicates only a minimum number of genes, the results computed with EqualTDRL weaken the support for the TDRL rearrangement that has been presented in [20] in favor of the TDRL rearrangement that is shown in Fig. 5. Moreover, the TDRL presented in Fig. 5 gets additional support by the detection of a putative remnant of the *trnM* gene in the intergenic region of *Lepidopsocidae sp.* between *trnQ* and *nad2*.

## Conclusion

In this article we have presented the tool EqualTDRL, which illustrates all equivalent TDRLs for a pair of gene orders that differ by one TDRL. EqualTDRL considers the circularity of genomes and helps to study their differences in consideration of all TDRL predictions that possibly could have taken place. Thereby, it helps to identify TDRLs that satisfy different biological constraints. For example, a requirement might be that the TDRL duplicates only a minimum number of genes or that the TDRL allows to explain the presence of intergenic regions. In addition, EqualTDRL supports scientists to determine whether the gene loss of a TDRL is random or might dependent on gene orientation or transcript structure. It has been shown for two example mitochondrial gene orders how EqualTDRL can be used to identify more plausible TDRLs that possibly could have taken place.

## Availability and requirements

**Project name:** EqualTDRL

**Project home page:** http://pacosy.informatik.uni-leipzig.de/equaltdrl

**Operating system(s):** Linux distribution

**Programming language:** C++,R

**Other requirements:** ggplot2 package of R

**License:** none

**Any restrictions to use by non-academics:** none

## Additional file

Additional file 1 Supplement. Additional file that contains the MITOS2 annotations and alignments of the mitochondrial genomes analyzed in the current study. (PDF 222 kb)

## Abbreviations

CR: Control region
TDRL: Tandem duplication random loss
