# Supplementary material for: EqualTDRL: illustrating equivalent tandem duplication random loss rearrangements
Source: BMC Bioinformatics. 2018 May 30;19:192. doi: 10.1186/s12859-018-2170-x (PMC5975268; doi:10.1186/s12859-018-2170-x)
Supplement: Supplementary file 1 — Supplement. Additional file that contains the MITOS2 annotations and alignments of the mitochondrial genomes analyzed in the current study. (PDF 222 kb) [file 12859_2018_2170_MOESM1_ESM.pdf]

SUPPLEMENTARY MATERIAL

EqualTDRL: illustrating equivalent tandem duplication random loss rearrangements Supplementary material

Tom Hartmann[1*], Matthias Bernt[2] and Martin Middendorf[1]

[*]Correspondence:
thartmann@informatik.uni-leipzig.de
[1]Swarm Intelligence and Complex Systems Group, Faculty of Mathematics and Computer Science, Leipzig University, Augustusplatz 10, D-04109 Leipzig, Germany
Full list of author information is available at the end of the article

Section 1 contains supplementary tables. Additional figures are given in Section 2.

1 Supplementary Tables

Table 1 MITOS2 annotation of the mitochondrial genome of Lepidopsocidae sp. with standard E-value exponent of 2. Putative gene duplications and parts of the control region are removed.

Gene	Start	End	Strand
trnQ	0	68	-
nad2	148	1039	+
trnC	1145	1212	-
trnY	1212	1277	-
cox1	1278	2790	+
trnL2	2816	2883	+
trnK	2879	2946	+
trnD	2946	3014	+
atp8	3014	3167	+
atp6	3163	3832	+
cox3	3840	4620	+
trnR	4625	4689	+
trnS1	4689	4757	+
trnE	4758	4824	+
trnS2	4823	4891	+
trnI	4892	4957	+
trnM	4957	5023	+
trnW	5025	5092	+
cox2	5093	5771	+
trnG	5777	5845	+
nad3	5842	6191	+
trnA	6204	6273	+
trnN	6272	6340	+
trnF	6340	6409	-
nad5	6455	8048	-
trnH	8162	8228	-
nad4	8193	9413	-
nad4l	9592	9790	-
trnT	9854	9919	+
trnP	9918	9986	-
nad6	10023	10467	+
cob	10494	11610	+
nad1	11669	12581	-
trnL1	12596	12664	-
rrnL	12643	13737	-
trnV	13950	14018	-
rrnS	14021	14773	-

Table 2 MITOS2 annotation of the mitochondrial genome of Lepidopsocidae sp. with E-value exponent of 1. Putative gene duplications and parts of the control region are removed.

Gene	Start	End	Strand
trnQ	0	68	-
nad2	157	1147	+
trnC	1145	1212	-
trnY	1212	1277	-
cox1	1269	2811	+
trnL2	2816	2883	+
trnK	2879	2946	+
trnD	2946	3014	+
atp8	3014	3170	+
atp6	3163	3841	+
cox3	3840	4626	+
trnR	4625	4689	+
trnS1	4689	4757	+
trnE	4758	4824	+
trnS2	4823	4891	+
trnI	4892	4957	+
trnM	4957	5023	+
trnW	5025	5092	+
cox2	5093	5774	+
trnG	5777	5845	+
nad3	5845	6199	+
trnA	6204	6273	+
trnN	6272	6340	+
trnF	6340	6409	-
nad5	6410	8132	-
trnH	8162	8228	-
nad4	8231	9566	-
nad4l	9559	9853	-
trnT	9854	9919	+
trnP	9918	9986	-
nad6	9987	10482	+
cob	10482	11625	+
nad1	11645	12575	-
trnL1	12596	12664	-
rrnL	12643	13737	-
trnV	13950	14018	-
rrnS	14021	14773	-

Table 3 MITOS2 annotation of the mitochondrial genome of Dorypteryx domestica with E-value exponent of 2. Putative gene duplications and parts of the control region are removed.

Gene	Start	End	Strand
trnQ	213	279	-
nad2	329	1319	+
trnC	1321	1383	-
trnY	1414	1480	-
cox1	1489	3022	+
trnL2	3024	3088	+
trnK	3085	3152	+
trnD	3151	3215	+
atp8	3215	3371	+
atp6	3364	4036	+
cox3	4039	4825	+
trnR	4823	4890	+
trnS1	4890	4950	+
trnE	4949	5029	+
trnS2	5008	5073	+
trnI	5078	5143	+
trnM	5143	5209	+
trnW	5210	5274	+
cox2	5278	5962	+
trnG	5961	6025	+
nad3	6025	6379	+
trnA	6377	6440	+
trnN	6439	6504	+
trnF	6529	6593	-
nad5	6597	8316	-
trnH	8316	8379	-
nad4	8384	9728	-
nad4l	9727	10006	-
trnT	10013	10075	+
trnP	10074	10139	-
nad6	10131	10623	+
cob	10626	11757	+
nad1	11778	12726	-
trnL1	12729	12794	-
rrnL	12771	14028	-
trnV	14052	14117	-
rrnS	14118	14841	-

Table 4 Homology detection results of tRNA and rRNA genes (`CMsearch` of Infernal software package [1]) and protein-coding genes (`HMMsearch` of `HMMER` 3.1b1 software package [2]) for the intergenic regions considered in the main text. Shown are only hits with an E-value that is smaller than 1. In the case of multiple hits, the best e-value is illustrated.

Species	Intergenic region	Gene	E-value	Bit-score
Lepidopsocidae sp.	trnQ...nad2	trnM	0.005	-3.28
Lepidopsocidae sp.	trnQ...nad2	trnS1	0.016	-2.98
Lepidopsocidae sp.	trnQ...nad2	nad6	0.21	2.5
Lepidopsocidae sp.	trnQ...nad2	atp6	0.23	2.2
Lepidopsocidae sp.	trnQ...nad2	nad2	0.29	1.0
Lepidopsocidae sp.	trnQ...nad2	nad4	0.42	0.3
Lepidopsocidae sp.	trnQ...nad2	nad5	0.51	-0.2
Lepidopsocidae sp.	trnQ...nad2	atp8	0.53	1.9
Lepidopsocidae sp.	trnQ...nad2	nad4l	0.59	1.5
Lepidopsocidae sp.	trnQ...nad2	nad1	0.97	-0.1

2 Supplementary Figures

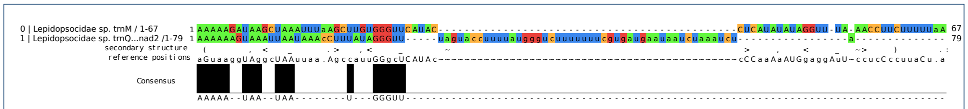

Figure 1 Local alignment of the intergenic region between trnQ and nad2 and the trnM gene of the mitochondrial genome of Lepidopsocidae sp. The alignment was generated with `CMalign` of Infernal software package [1].

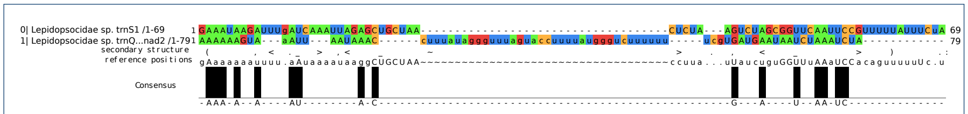

Figure 2 Local alignment of the intergenic region between trnQ and nad2 and the trnS1 gene of the mitochondrial genome of Lepidopsocidae sp. The alignment was generated with `CMalign` of Infernal software package [1].

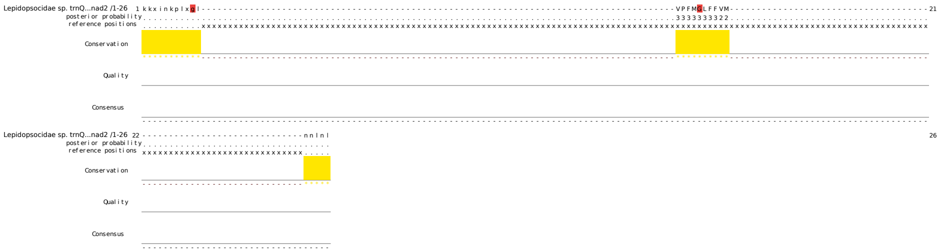

Figure 3 Alignment of the intergenic region between trnQ and nad2 and the nad6 gene of the mitochondrial genome of Lepidopsocidae sp. The alignment was generated with `HMMalign` of `HMMER` 3.1b1 software package [2].

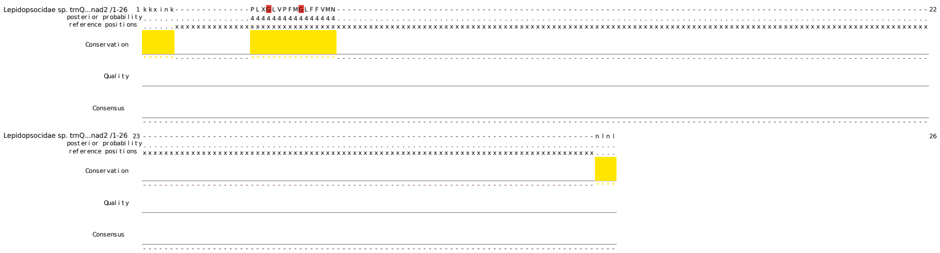

Figure 4 Alignment of the intergenic region between trnQ and nad2 and the atp6 gene of the mitochondrial genome of Lepidopsocidae sp. The alignment was generated with `HMMalign` of `HMMER` 3.1b1 software package [2].

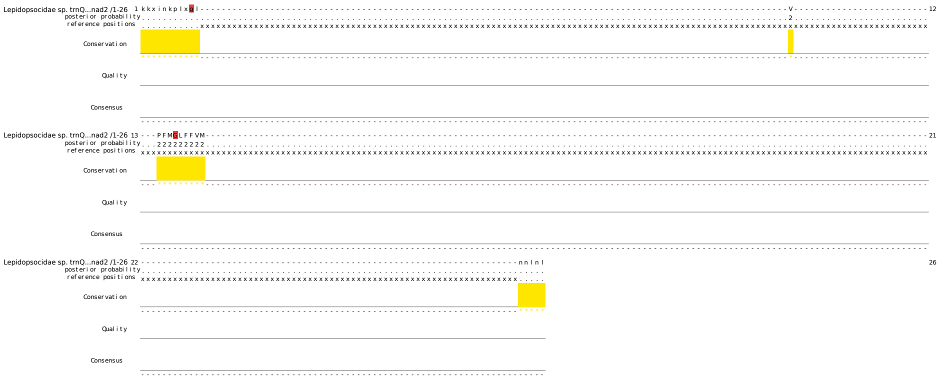

Figure 5 Alignment of the intergenic region between trnQ and nad2 and the nad2 gene of the mitochondrial genome of Lepidopsocidae sp. The alignment was generated with `HMMalign` of `HMMER` 3.1b1 software package [2].

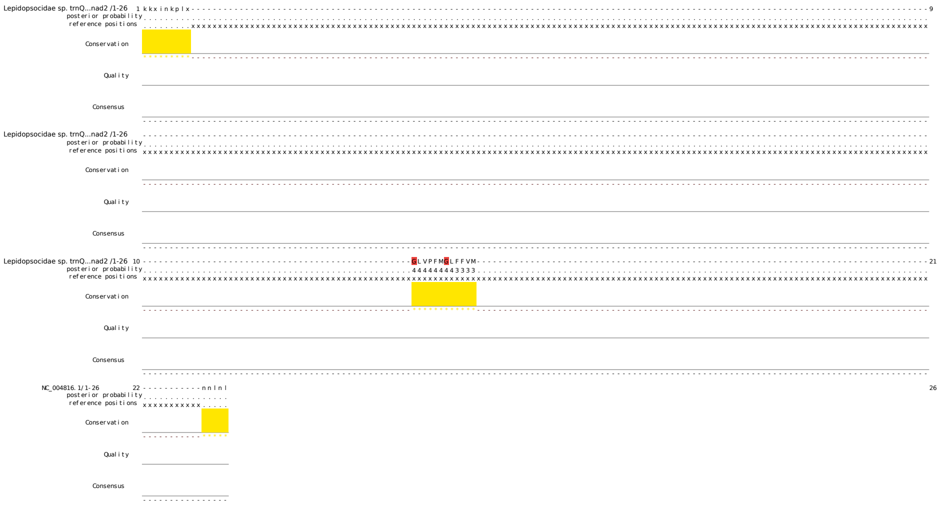

Figure 6 Alignment of the intergenic region between trnQ and nad2 and the nad4 gene of the mitochondrial genome of Lepidopsocidae sp. The alignment was generated with `HMMalign` of `HMMER` 3.1b1 software package [2].

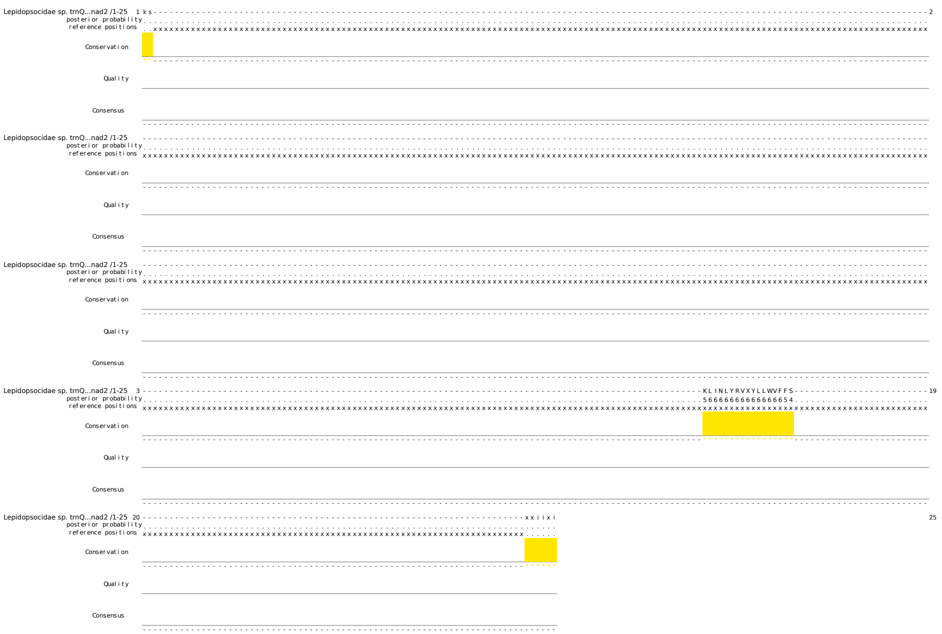

Figure 7 Alignment of the intergenic region between trnQ and nad2 and the nad5 gene of the mitochondrial genome of Lepidopsocidae sp. The alignment was generated with `HMMalign` of `HMMER` 3.1b1 software package [2].

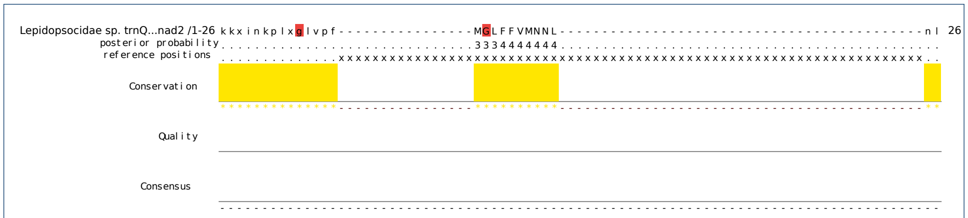

Figure 8 Alignment of the intergenic region between trnQ and nad2 and the atp8 gene of the mitochondrial genome of Lepidopsocidae sp. The alignment was generated with `HMMalign` of `HMMER` 3.1b1 software package [2].

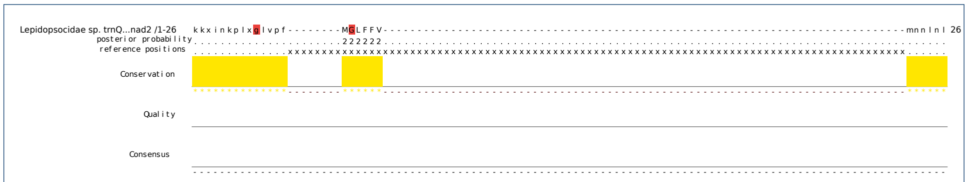

Figure 9 Alignment of the intergenic region between trnQ and nad2 and the nad4l gene of the mitochondrial genome of Lepidopsocidae sp. The alignment was generated with `HMMalign` of `HMMER` 3.1b1 software package [2].

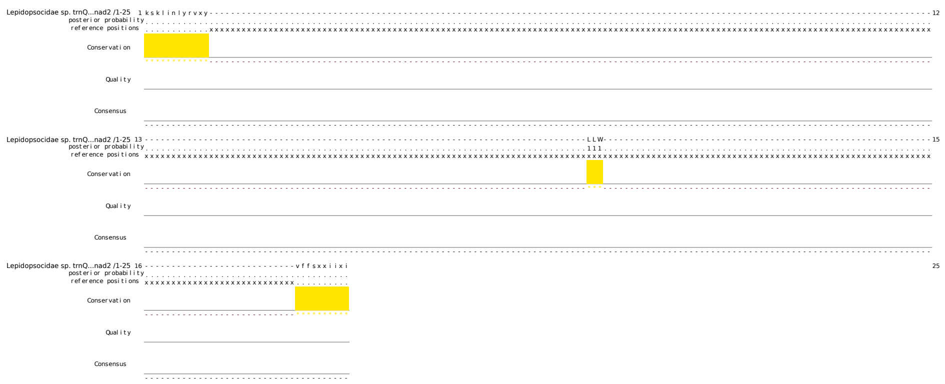

Figure 10 Alignment of the intergenic region between trnQ and nad2 and the nad1 gene of the mitochondrial genome of Lepidopsocidae sp. The alignment was generated with `HMMalign` of `HMMER` 3.1b1 software package [2].

Author details
[1]Swarm Intelligence and Complex Systems Group, Faculty of Mathematics and Computer Science, Leipzig University, Augustusplatz 10, D-04109 Leipzig, Germany. [2]Helmholtz Centre for Environmental Research - UFZ, Permoserstraße 15, D-04318 Leipzig, Germany.

References

1. Nawrocki, E.P., Eddy, S.R.: Infernal 1.1: 100-fold faster RNA homology searches. Bioinformatics **29**(22), 2933–2935 (2013)
2. Finn, R.D., Clements, J., Eddy, S.R.: HMMER web server: interactive sequence similarity searching. Nucleic acids research **39**(suppl_2), 29–37 (2011)
